# Therapeutic effect of *Schistosoma japonicum* cystatin on bacterial sepsis in mice

**DOI:** 10.1186/s13071-017-2162-0

**Published:** 2017-05-08

**Authors:** Huihui Li, Shushu Wang, Bin Zhan, Wenxin He, Liang Chu, Dapeng Qiu, Nan Li, Yongkun Wan, Hui Zhang, Xingzhi Chen, Qiang Fang, Jilong Shen, Xiaodi Yang

**Affiliations:** 1 Basic Medical College of Bengbu Medical College, Bengbu, 233000 China; 2 Anhui Key Laboratory of Infection and Immunity of Bengbu Medical College, Bengbu, 233000 China; 3 Pediatrics Department of Affiliated Provincial Hospital of Anhui Medical University, Hefei, 230001 China; 4 Section of Tropical Medicine, Department of Pediatrics, Baylor College of Medicine, Houston, TX USA; 5 Second Affiliated Hospital of Bengbu Medical College, Bengbu, 233000 China; 6 Department of Immunology, Anhui Medical University, Hefei, 230022 China

**Keywords:** Cystatin, *Schistosoma japonicum*, Sepsis, Cecal ligation and puncture, Immunomodulation

## Abstract

**Background:**

Sepsis is a life-threatening complication of an infection and remains one of the leading causes of mortality in surgical patients. Bacteremia induces excessive inflammatory responses that result in multiple organ damage. Chronic helminth infection and helminth-derived materials have been found to immunomodulate host immune system to reduce inflammation against some allergic or inflammatory diseases. *Schistosoma japonicum* cystatin (*Sj*-Cys) is a cysteine protease inhibitor that induces regulatory T-cells and a potential immunomodulatory. The effect of *Sj*-Cys on reducing sepsis inflammation and mortality was investigated.

**Methods:**

Sepsis was induced in BALB/c mice using cecal ligation and puncture (CLP), followed by intraperitoneal injection of different doses (10, 25 or 50 μg) of recombinant *Sj*-Cys (r*Sj*-Cys). The therapeutic effect of r*Sj*-Cys on sepsis was evaluated by observing the survival rates of mice for 96 h after CLP and the pathological injury of liver, kidney and lung by measuring the levels of alanine transaminase (ALT), aspartate transaminase (AST), blood urea nitrogen (BUN) and creatinine (Cr) in sera and the tissue sections pathology, and the expression of MyD88 in liver, kidney and lung tissues. The immunological mechanism was investigated by examining pro-inflammatory cytokines (TNF-α, IL-6, IL-1β) and IL-10 and TGF-β1 in mice sera and in culture of macrophages stimulated by lipopolysaccharides (LPS).

**Results:**

r*Sj*-Cys treatment provided significant therapeutic effects on CLP-induced sepsis in mice demonstrated with increased survival rates, alleviated overall disease severity and tissue injury of liver, kidney and lung. The r*Sj*-Cys conferred therapeutic efficacy was associated with upregualted IL-10 and TGF-β1 cytokines and reduced pro-inflammatory cytokines TNF-α, IL-6, IL-1β. MyD88 expression in liver, kidney and lung tissues of r*Sj*-Cys-treated mice was reduced. In vitro assay with macrophages also showed that r*Sj*-Cys inhibited the release of pro-inflammatory cytokines and mediator nitric oxide (NO) after being stimulated by lipopolysaccharide (LPS).

**Conclusions:**

The results suggest the anti-inflammatory potential of r*Sj*-Cys as a promising therapeutic agent on sepsis. The immunological mechanism underlying its therapeutic effect may involve the downregulation of pro-inflammatory cytokines and upregulation of IL-10 and TGF-β1 cytokines possibly via downregulation of the TLR adaptor-transducer MyD88 pathway. The findings suggest r*Sj*-Cys is a potential therapeutic agent for sepsis and other inflammatory diseases.

## Background

Sepsis is one of most serious complications of clinical critical patients with infections [1, 2]. Although extensive research has made some progress in several fields of sepsis, the appropriate therapeutic intervention is limited and sepsis is still associated with high morbidity and mortality worldwide [3, 4]. In sepsis, bacterial lipopolysaccharide (LPS) endotoxin induces macrophages and other effective cells to release massive pro-inflammatory cytokines which play pivotal roles in the development of systemic inflammatory responses [5, 6]. The excessive inflammatory responses damage the structure and function of vital organs such as kidney, liver and lung [7–11], and even lead to multiple organ dysfunction syndromes (MODS) and death. Therefore, how to inhibit the release of pro-inflammatory cytokines and balance immune response has been suggested as an important strategy to treat sepsis and reduce its mortality.

Helminthic infections trigger Th2-skewed immune responses characterized by activated Th2 cells and related cytokines including high levels of IgE, IgG1 and eosinophils [12]. During infection in host, helminth worms secret a variety of molecules to modulate host immune responses as a strategy to escape host immune attack [13]. The immunomodulatory effect usually takes effect through stimulation of regulatory T cells (Tregs) characterized by increased level of IL-10, TGF-β1 and FoxP3^+^ T cells [14]. The effect of immunomodulation protects worms from being attacked by host immune defense; on the other hand it balances host immune system to reduce susceptibility to autoimmune or allergic diseases caused usually by hypersensitivity to endogenous self-antigens or exogenous allergens [15]. This concept has been adopted to use helminth infection or helminth-derived proteins to treat a variety of allergic or autoimmune diseases and significant relief of these diseases has been observed [16–18].

Helminths secrete various cysteine protease inhibitors called cystatins [19] which play important roles in escaping host immune attack by directly inhibiting host cysteine proteases or suppressing host immune response through regulating inflammatory cytokines [20–28] or inhibiting class II MHC-restricted antigen processing and antigen epitope presentation [22], therefore cystatins from various parasitic helminths are being explored as potential therapeutic agents for allergic diseases or immunological disorders including allergic asthma [26], inflammatory colitis [27, 29], and collagen-induced arthritis [30]. The cystatin from the blood-feeding trematode *Schistosoma japonicum* (*Sj*-Cys) has been identified not only to inhibit the proteolytic activity of cysteine protease papain [31], but also to inhibit the release of pro-inflammatory cytokines such as TNF-α and IL-6 from RAW264.7 induced by LPS in vitro [25]. Whether *Sj*-Cys inhibits the release of pro-inflammatory cytokines during serious infection and whether *Sj*-Cys can be used as a therapeutic agent for sepsis have not been investigated so far.

In the present study, we investigated the therapeutic effect of recombinant *Sj*-Cys (r*Sj*-Cys) on sepsis using a cecal ligation and puncture (CLP)-induced sepsis mice model. We identified that the administration of r*Sj*-Cys significantly reduced the mortality of CLP-induced sepsis and its therapeutic effect took effect through inhibiting the pro-inflammatory cytokines and boosting IL-10 and TGF-β1 cytokines, providing a promising strategy for sepsis therapy in clinical practice.

## Methods

### Production of recombinant *Sj*-Cys protein

DNA coding for full-length *Sj*-Cys (GenBank: CAX73577.1) was amplified from total cDNA of *S. japonicum* adult worms using following primers (forward: 5'-CAG AAT TCA TGC CTT TAT GTT GTG GTG GT G-3'; reverse: 5'-GCC TCG AGT TAG AAA TAA TAG AAA TGT AAC AGC-3') and cloned into pET-28a (+) (Promega, Madison, Wisconsin, USA) using EcoRI and XhoI restriction sites. The sequencing-confirmed recombinant plasmid was transformed into *E. coli* BL 21. The expression of r*Sj*-Cys was induced with 1 mM isopropylthio-β-galactoside (IPTG, Sigma-Aldrich, Steinheim, Germany) at 37 °C for 5 h. The expressed r*Sj*-Cys with His-tag at N-terminus was purified with a Ni–NTA His* Bind Purification Kit (Merck Millipore, Basilica, Massachusetts, USA). The contaminated endotoxin in purified r*Sj*-Cys was removed by using a ToxOut™ High Capacity Endotoxin Removal Kit (BioVision, Palo Alto, California, USA) and detected by ToxinSensor™ Chromogenic Limulus Amebocyte Lysate (LAL) Endotoxin Assay Kit (GenScript Biotechnology, Nanjing, China) following the manufacturer’s protocol. The concentration of r*Sj*-Cys was measured using Bicinchoninic Acid Protein Assay Kit (Beyotime Biotechnology, Shanghai, China).

### Preparation and stimulation of murine peritoneal exudate cells

Donor BALB/c mice were euthanized and the murine peritoneal exudate cells (PECs) were collected by washing the peritoneal cavity with 5 ml of ice-cold phosphate-buffered saline (PBS) supplemented with 2% heat-inactivated fetal bovine serum (FBS) (Zhejiang Tianhang Biological Technology, Zhejiang, China) as described in [32]. The recovered cells were washed three times with PBS and resuspended in RPMI 1640 medium supplemented with 10% FBS, 100 U/ml penicillin and 100 μg/ml streptomycin (Sigma-Aldrich, Steinheim, Germany). The cells were coated on 24-well plate (2 × 10^6^ cells/well in 1 ml) at 5% CO_2_, 37 °C for 4 h. The non-adherent cells were removed by washing with RPMI 1640 and the adherent cells were stimulated with LPS (2 μg/ml) (Sigma-Aldrich, Steinheim, Germany, USA) in the presence of r*Sj*-Cys (2 μg/ml) for 24 h. The concentrations of TNF-α, IL-6, IL-1β in the culture supernatants were measured using LEGEND MAX™ ELISA kits (Dakewe Biotech, Beijing, China) and nitric oxide (NO) was measured by nitrate reductase method using NO assay kit (Nanjing Jiancheng Bio-engineering Institute, Nanjing, China) according to the manufacturer’s instruction.

### Animals and cecal ligation and puncture-induced sepsis

Male BALB/c mice (specific pathogen free) with 6–8 weeks old and weighing 18–22 g, were purchased from the Animal Center of Anhui Medical University and maintained in a controlled environment (12:12 h light/dark cycle with a temperature of 22 ± 2 °C and a relative humidity of 55%). Water and food were provided ad libitum.

A clinically relevant rodent model of sepsis was created by cecal ligation and puncture (CLP) based on the method described in [3]. Briefly, mice were fasted for 12 h with drinking water only and then anaesthetized by intraperitoneal injection of chloral hydrate (40 g/l) 0.2 ml/20 g. The abdominal cavity was opened with a midline incision in layers. The cecum was isolated and ligated 1.0 cm from the tip. A through-and-through puncture was made with an 18-gauge needle and a small amount of feces was extruded to ensure the patency of the puncture site before returning the cecum back to the abdominal cavity. Each layer opened was closed with suture. The control mice underwent a sham surgery receiving a laparotomy without cecal ligation and puncture. The general condition and survival rate of mice were observed for the following 4 days.

### Treatment of CLP-induced sepsis with r*Sj*-Cys

Mice were divided into 5 groups, four groups of 16 mice each were CLP operated and treated intraperitoneally with 10 μg, 25 μg and 50 μg of r*Sj*-Cys or PBS in a total volume of 200 μl 30 min after surgery, respectively. Another group of 16 mice were performed sham surgery without ligation and puncture of the cecum and given with PBS only. Six mice in each group were sacrificed 12 h after surgery for measuring inflammatory cytokines and biomarkers of tissue injury in blood and pathology of liver, kidney and lung. The remaining 10 mice were observed for general physical conditions and survival rate for 96 h. The survival rates were determined using Kaplan-Meier method.

### Serological evaluation of sepsis-caused liver and kidney injury and cytokine changes

Blood samples were collected 12 h after surgery, and sera were saved for measuring the levels of alanine transaminase (ALT), aspartate transaminase (AST), blood urea nitrogen (BUN) and creatinine (Cr) by automatic chemistry analyzer (Beckman Coulter, Brea, California, USA) to evaluate sepsis-caused liver and kidney injury. The serum levels of pro-inflammatory cytokines (TNF-α, IL-6 and IL-1β) and IL-10 and TGF-β1 cytokines were measured using LEGEND MAX™ ELISA kits (Dakewe Biotech, Beijing, China) following the manufacturer’s instructions.

### Macroscopic and histopathological changes in liver, kidney and lung

The liver, kidney and lung tissues were collected from each mice 12 h after surgery, and fixed with 4% paraformaldehyde. The tissue sections were stained with hematoxylin and eosin and observed under a microscope (Olympus, Tokyo, Japan). The histological liver injury was scored based on hepatocellular necrosis, bleeding, and inflammatory cell infiltration in the liver [33] as shown in Table 1. The histological kidney injury was scored based on the injured renal tubules and shrunk glomerulus [34, 35] as shown in Table 2. The histological lung injury was scored based on the alveolar congestion, hemorrhage, neutrophil infiltration into the airspace or vessel wall, and thickness of alveolar wall/hyaline membrane formation [36] as shown in Table 3. Histopathological changes in the liver, kidney and lung tissues were blindly reviewed and scored as described above.

**Table 1:** Liver injury score parameters

Index	0	1	2	3	Maximum
Necrosis	None	Focal piecemeal	Continuous < 50%	Continuous > 50%	3
Bleeding	None	<30%	30–50%	>50%	3
Infiltration	None	2- to 3-fold	3- to 10-fold	>10-fold	3

**Table 2:** Kidney injury score parameters

Injured renal tubules and shrunk glomerulus	Index
None	0
<10%	1
11–25%	2
26–45%	3
46–75%	4
>76%	5

**Table 3:** Lung injury score parameters

Changes of lung tissues structure	Index
None	1
Focal interstitial congestion and inflammatory cell infiltration < 50%	2
Diffuse interstitial congestion and inflammatory cell infiltration > 50%	3
Focal consolidation and inflammatory cell infiltration < 50%	4
Diffuse consolidation and inflammatory cell infiltration > 50%	5

### Western blot detection of MyD88 in liver, kidney and lung tissues of mice

The liver, kidney and lung tissues of mice were homogenized and separated by 12% polyacrylamide gel electrophoresis. Proteins were blotted onto a 0.45 μm polyvinylidene fluoride (PVDF) membrane. Rabbit anti-MyD88 monoclonal antibody (Cell Signaling Technology, Danvers, Massachusetts, USA) (1:1,000) and rabbit anti-β-actin polyclonal antibody (Cell Signaling Technology, Danvers, Massachusetts, USA) (1:2,000) were used to probe MyD88 and β-actin in mice tissues. HRP-conjugated goat anti-rabbit IgG (Merck Millipore, Basilica, Massachusetts, USA) was used as secondary antibody at 1:10,000 dilution. The density of recognized MyD88 was compared to that of control β-actin and their ratio was used as a relative expression level of MyD88 in injured mice tissues.

### Statistical analysis

All data were presented as the mean ± SEM (standard error of the mean), and the statistical analyses were performed using SPSS 16.0 software. Comparison of the same parameters in multiple datasets or more than two groups was done using one-way analysis of variance (ANOVA). The difference in survival rates among the groups was compared using Kaplan-Meier survival analysis. *P* < 0.05 was considered as statistically significant.

## Results

### Expression, purification and identification of r*Sj*-Cys

**Fig. 1:**
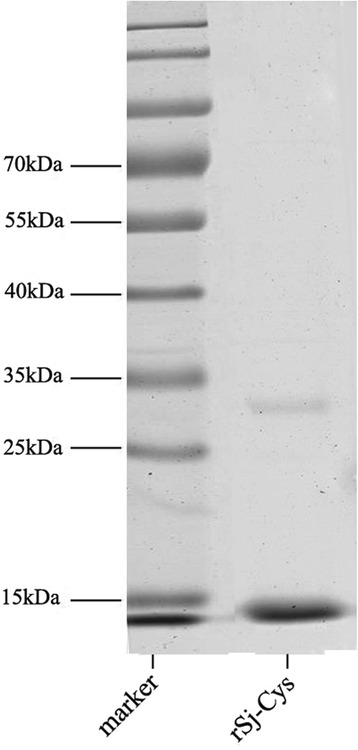
SDS-PAGE of purified r*Sj*-Cys. Total 2 μg of purified r*Sj*-Cys was separated by 12% polyacrylamide gel electrophoresis

The r*Sj*-Cys was successfully expressed in *E. coli* as soluble recombinant protein. SDS-PAGE showed that the purified r*Sj*-Cys appeared as size of about 11 kDa, which corresponds well to the molecular weight of deduced peptide gene product (11.3 kDa) (Fig. 1). The contaminated endotoxin level in purified r*Sj*-Cys was as less than 0.06 EU/ml after running through the endotoxin removal column.

### The inhibitory effects of r*Sj*-Cys on murine peritoneal exudate cells upon LPS stimulation

**Fig. 2:**
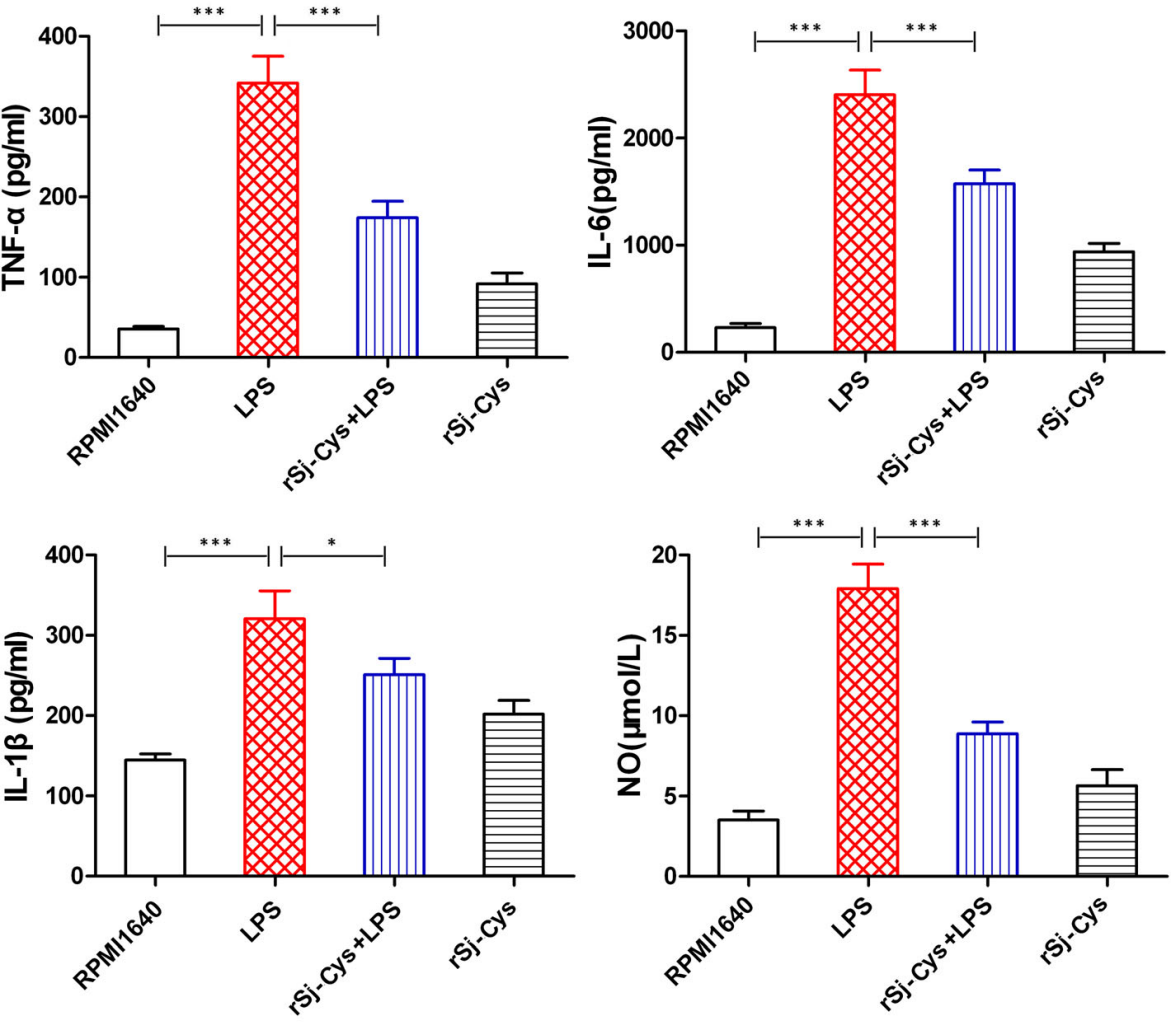
r*Sj*-Cys reduces the releasing of pro-inflammatory cytokines and nitrous oxide from murine peritoneal exudate cells stimulated with LPS. The adherent peritoneal exudate cells were cultured and stimulated with LPS in the presence or absence of r*Sj*-Cys. The levels of TNF-α, IL-6, IL-1β and NO were measured in culture supernatants collected after 24 h incubation. The results are shown as the mean ± SEM for each group. *P < 0.05, ***P < 0.001

Upon stimulation of LPS at concentration of 2 μg/ml, adherent murine peritoneal exudate cells (PECs), mostly macrophages, secreted high levels of pro-inflammatory cytokines including TNF-α, IL-6 and IL-1β (ANOVA: *F*_(3,23)_ = 41.00, *P* < 0.0001; *F*_(3,23)_ = 46.04, *P* < 0.0001; *F*_(3,23)_ = 11.70, *P* < 0.0001, respectively) and released more inflammatory mediator nitrous oxide (NO) (ANOVA: *F*_(3,23)_ = 39.02, *P* < 0.0001) compared to cells incubated with RPMI 1640 alone. The addition of r*Sj*-Cys (2 μg/ml) significantly inhibited PECs to secrete TNF-α, IL-6, IL-1β and NO upon stimulation of LPS compared to cells in LPS stimulation without r*Sj*-Cys (ANOVA: *F*_(3,23)_ = 41.00, *P* < 0.0001; *F*_(3,23)_ = 46.04, *P* < 0.0001; *F*_(3,23)_ = 11.70, *P* < 0.0001; *F*_(3,23)_ = 39.02, *P* < 0.0001, respectively) (Fig. 2), indicating that r*Sj*-Cys suppresses the macrophages pro-inflammatory release to LPS stimulation. r*Sj*-Cys itself stimulated low level of TNF-α, IL-6, IL-1β and NO released by PECs.

### r*Sj*-Cys ameliorates CLP-induced sepsis

**Fig. 3:**
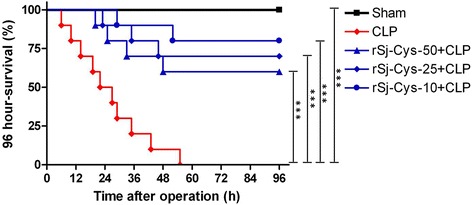
r*Sj*-Cys treatment reduced mortality of mice with sepsis induced by CLP. After CLP surgery, mice were injected intraperitoneally with different doses of r*Sj*-Cys. Mice with sham surgery and treated with PBS were used as control. The survival rate was determined using Kaplan-Meier method and compared by log-rank test (*n* = 10 mice per group). ***P < 0.001

r*Sj*-Cys was used to treat CLP-induced sepsis in a mouse model to determine whether it is able to ameliorate sepsis. As shown in Fig. 3, CLP induced severe sepsis with all mice died 55 h after surgery without treatment (Kaplan-Meier analysis compared with sham group: *χ* ^2^ = 21.84, *df* = 1, *P* < 0.0001). However, mice treated with r*Sj*-Cys 30 min after the CLP surgery significantly reduced their mortality caused by sepsis. Compared to mice with sepsis without treatment, mice treated with 10 μg r*Sj*-Cys had a survival rate of 80% 96 h after CLP surgery (Kaplan-Meier analysis: *χ* ^2^ = 16.63, *df* = 1, *P* < 0.0001) while mice treated with 25 μg or 50 μg of r*Sj*-Cys had a survival rate of 70% (Kaplan-Meier analysis: *χ* ^2^ = 13.23, *df* = 1, *P* = 0.0003) and 60% (Kaplan-Meier analysis: *χ* ^2^ = 9.32, *df* = 1, *P* = 0.0023), respectively (Fig. 3). Mice with sham surgery all survived for 96 h period.

### r*Sj*-Cys inhibits pro-inflammatory cytokines and induces IL-10 and TGF-β1 in mice with CLP-induced sepsis

**Fig. 4:**
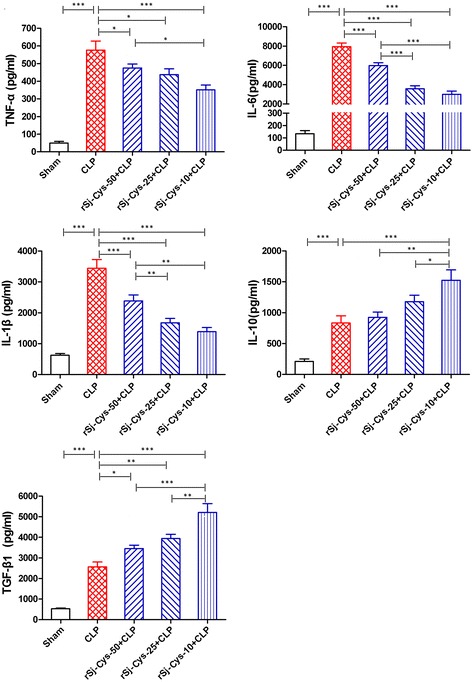
r*Sj*-Cys reduced the inflammatory cytokines (TNF-α, IL-6, IL-1β) and induced IL-10 and TGF-β1 releasing in mice with CLP-induced sepsis. The levels of these cytokines in sera of mice were measured by ELISA 12 h after the surgery. The results are shown as the mean ± SEM for each group (*n* = 6). *P < 0.05, **P < 0.01, ***P < 0.001

Sepsis is characterized by surged pro-inflammatory cytokines in circulating blood [37]. We examined the serum levels of some pro-inflammatory cytokines in mice with sepsis treated with or without r*Sj*-Cys to investigate whether r*Sj*-Cys rescues mice with sepsis through inhibiting inflammatory cytokine storm. The results demonstrated that serum levels of the pro-inflammatory cytokines (TNF-α, IL-6 and IL-1β) were dramatically increased in mice with sepsis 12 h after CLP surgery compared to mice with sham surgery (ANOVA: *F*_(4,29)_ = 40.17, *P* < 0.0001; *F*_(4,29)_ = 97.86, *P* < 0.0001; *F*_(4,29)_ = 36.25, *P* < 0.0001, respectively). The injection of r*Sj*-Cys significantly reduced the production of inflammatory cytokines including TNF-α, IL-6 and IL-1β compared to mice with sepsis without treatment (Fig. 4). r*Sj*-Cys at dose of 10 μg had the better inhibitory effects on the release of TNF-α, IL-6 and IL-1β compared to the doses of 50 μg (ANOVA: *F*_(4,29)_ = 40.17, *P* < 0.0001; *F*_(4,29)_ = 97.86, *P* < 0.0001; *F*_(4,29)_ = 36.25, *P* < 0.0001, respectively), consistent with the survival results that showed the treatment with r*Sj*-Cys at 10 μg dose had the highest survival rate (Fig. 3). The treatment with r*Sj*-Cys promoted secretion of IL-10 and TGF-β1 in sera, and the treatment with r*Sj*-Cys at dose of 10 μg had the highest secretion of IL-10 and TGF-β1 compared to the doses of 25 μg and 50 μg (ANOVA: *F*_(4,29)_ = 19.30, *P* < 0.0001; *F*_(4,29)_ = 49.44, *P* < 0.0001) (Fig. 4). These data indicate that r*Sj*-Cys rescues mice from lethal sepsis through inhibiting systemic inflammatory cytokines possibly through stimulating the secretion of regulatory cytokines such as IL-10 and TGF-β1.

### r*Sj*-Cys reduces pathology caused by CLP-induced sepsis

As r*Sj*-Cys decreased systemic inflammatory cytokines, we examined whether treatment with r*Sj*-Cys prevented injury of some important organs caused by CLP-induced sepsis.

#### Liver

**Fig. 5:**
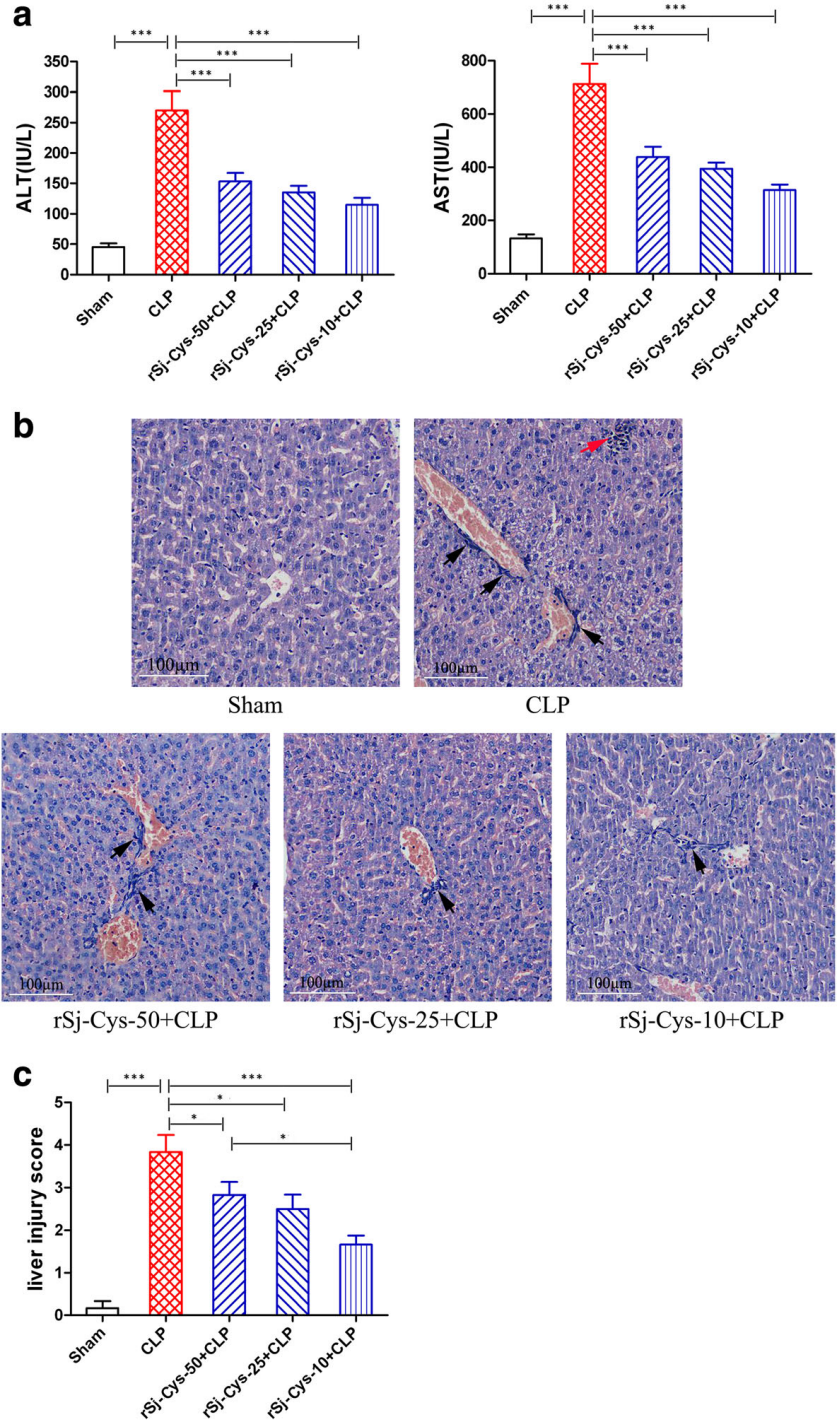
r*Sj*-Cys reduced liver injury caused by CLP-induced sepsis. **a** The serum levels of ALT and AST were reduced in mice treated with r*Sj*-Cys. **b** Representative liver sections showing reduced hepatocytes swelling and inflammatory cell infiltration in r*Sj*-Cys treated mice (×200; *Scale-bars*: 100 μm) (*red arrow*: hepatocellular necrosis; *black arrow*: inflammatory cell) and the improved liver injury score in r*Sj*-Cys treated mice (**c**). The results are shown as the mean ± SEM for each group (*n* = 6). *P < 0.05, ***P < 0.001

The serum levels of ALT, AST were greatly increased of mice with CLP-induced sepsis compared to mice with sham surgery (ANOVA: *F*_(4,29)_ = 22.39, *P* < 0.0001; *F*_(4,29)_ = 26.26, *P* < 0.0001, respectively), reflecting the serious injury of liver cells (Fig. 5a). Histopathology of liver tissues of mice with sepsis revealed hepatic cords disorder, hepatocytes swelling, inflammatory cell infiltration (Fig. 5b). The liver injury scores was significantly increased in mice with sepsis compared to mice with sham surgery (ANOVA: *F*_(4,29)_ = 21.31, *P* < 0.0001) (Fig. 5c). After being treated with r*Sj*-Cys, the serum levels of ALT, AST were obviously decreased, and the sepsis-caused liver injury scores was much reduced with the best results for group treated with 10 μg of r*Sj*-Cys compared to mice with sepsis without treatment, which is consistent with the highest levels of IL-10 and TGF-β1 cytokines and lowest levels of pro-inflammatory cytokines in sera measured above.

#### Kidney

**Fig. 6:**
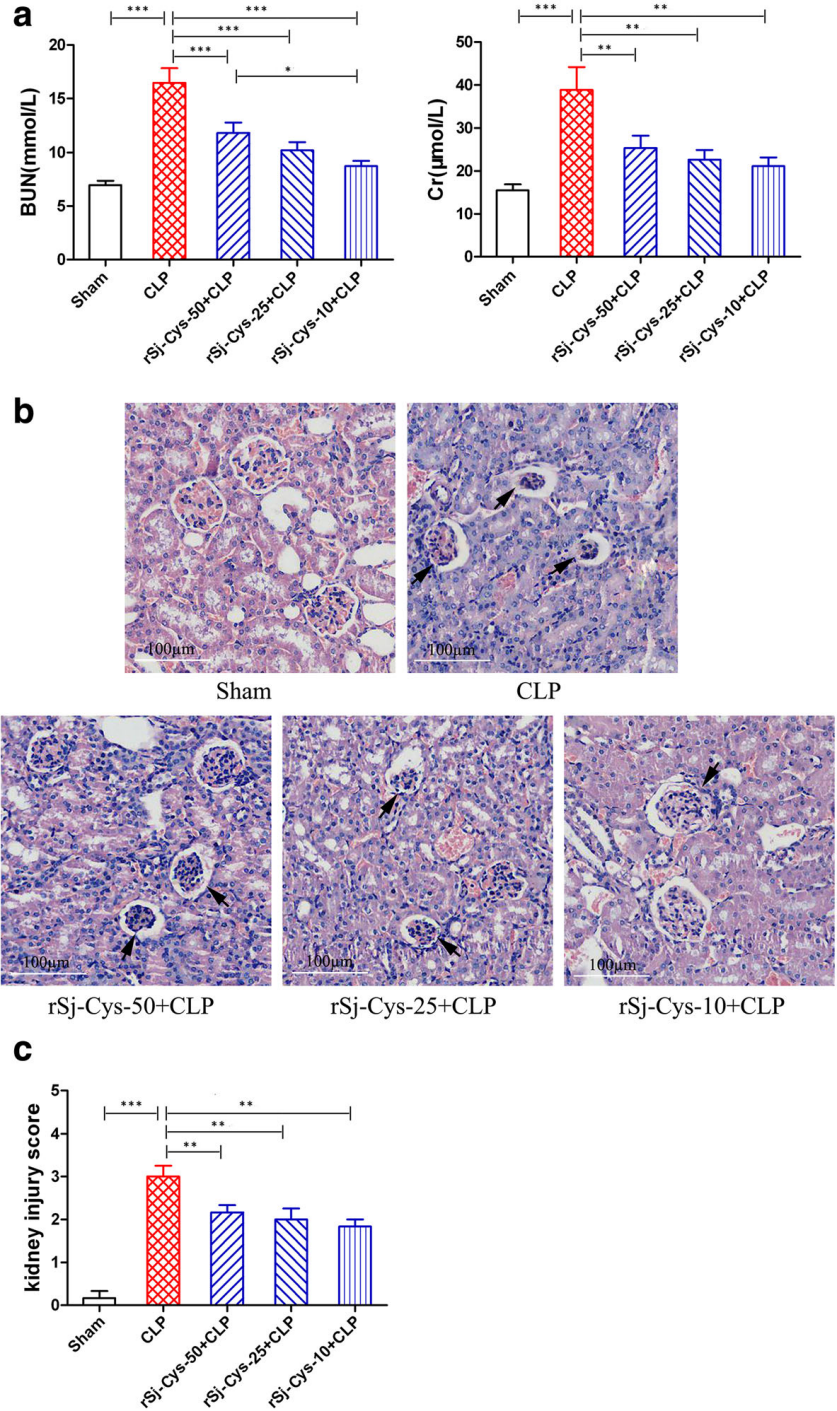
r*Sj*-Cys reduced kidney injury caused by CLP-induced sepsis. **a** The serum levels of BUN and Cr were reduced in mice treated with r*Sj*-Cys. **b** Representative kidney sections showing reduced renal tissue disrupture and inflammatory cell infiltration in r*Sj*-Cys treated mice (×200; *Scale-bars*: 100 μm) (arrows indicate shrunk glomerulus) and the improved kidney injury score (**c**). The results are shown as the mean ± SEM for each group (*n* = 6). *P < 0.05, **P < 0.01, ***P < 0.001

The serum levels of BUN and Cr were also highly increased in mice with CLP-induced sepsis compared to mice with sham surgery (ANOVA: *F*_(4,29)_ = 17.48, *P* < 0.0001; *F*_(4,29)_ = 7.78, *P* = 0.0003, respectively), indicating the serious injury of kidney cells (Fig. 6a). The kidney histopathology results revealed that some of glomerulus disrupted and distorted, renal tubular cells edema, plenty of infiltrated inflammatory cells (Fig. 6b) and kidney injury scores was significantly increased in mice with sepsis compared to mice with sham surgery (ANOVA: *F*_(4,29)_ = 24.68, *P* < 0.0001) (Fig. 6c). After treatment with r*Sj*-Cys, the serum levels of BUN and Cr were significantly decreased compared to mice with sepsis without treatment, the sepsis-caused kidney injury was mitigated and kidney injury scores was much reduced (ANOVA: *F*_(4,29)_ = 24.68, *P* < 0.0001) (Fig. 6b, c).

#### Lung

**Fig. 7:**
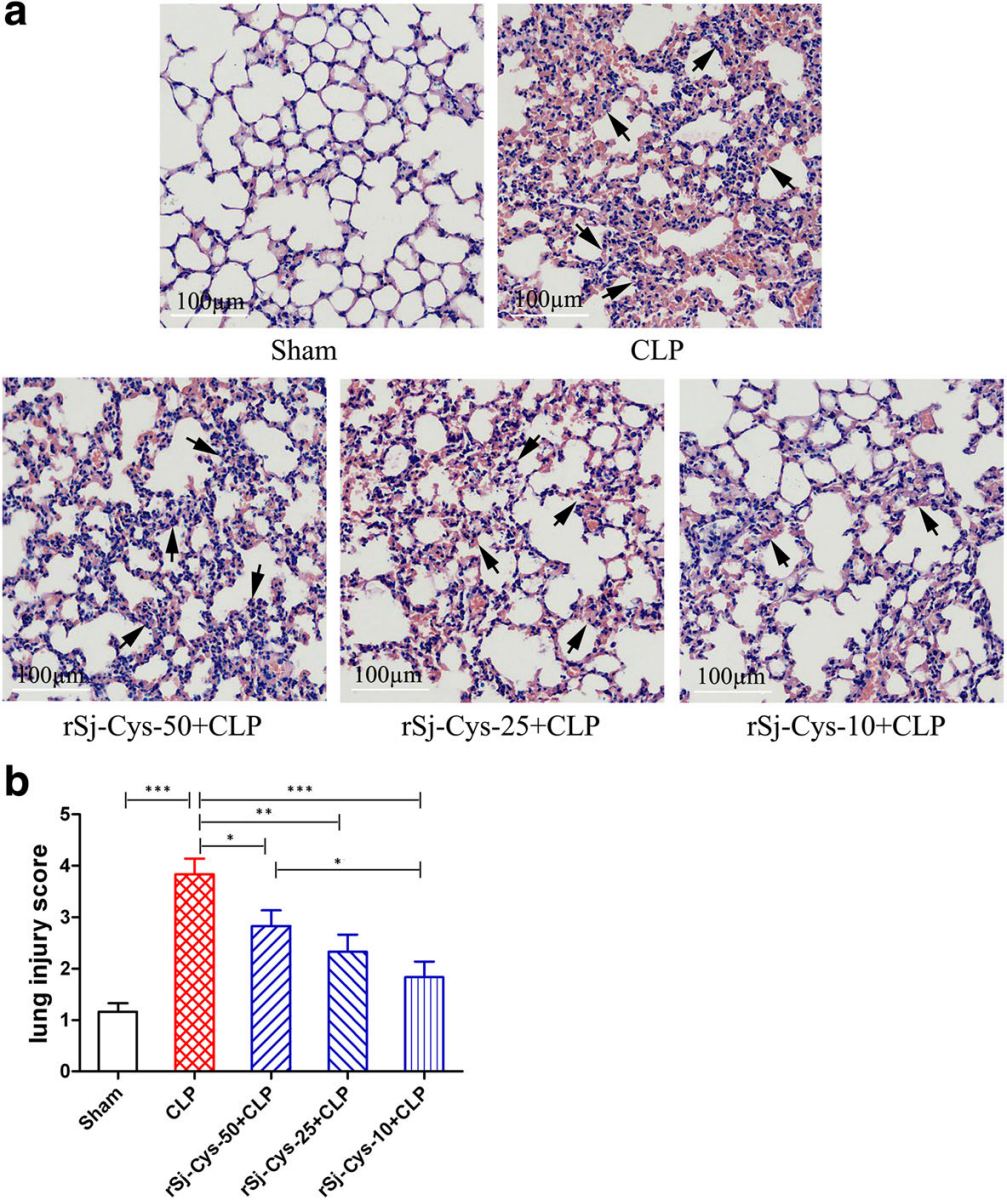
r*Sj*-Cys reduced lung injury caused by CLP-induced sepsis. **a** Representative lung tissue sections showing reduced alveolar structural disruption and reduced inflammatory cell infiltration in r*Sj*-Cys treated mice (×200; *Scale-bars*: 100 μm) (arrows indicate interalveolar septum thickened). **b** The improved lung injury score based on the tissue injury. The results are shown as the means ± SEM for each group (*n* = 6). *P < 0.05, **P < 0.01, ***P < 0.001

The lung sections of mice in sham group exhibited normal lung architecture with no evidence of inflammation, however the lung sections of mice with sepsis showed that the alveolar structural injury, interalveolar septum thickened and disrupted, inflammatory cell infiltration (Fig. 7a) and lung injury scores was significantly increased in mice with sepsis compared to mice with sham surgery (ANOVA: *F*_(4,29)_ = 12.11, *P* < 0.0001) (Fig. 7b). After treatment with r*Sj*-Cys, the sepsis-caused lung injury was improved and lung injury scores was much reduced compared to mice with sepsis without treatment, with the best results in group treated with 10 μg of r*Sj*-Cys, which is consistent with the highest levels of IL-10 and TGF-β1 cytokines and lowest levels of pro-inflammatory cytokines in sera.

### r*Sj*-Cys suppressed the expression of MyD88 with CLP-induced sepsis

**Fig. 8:**
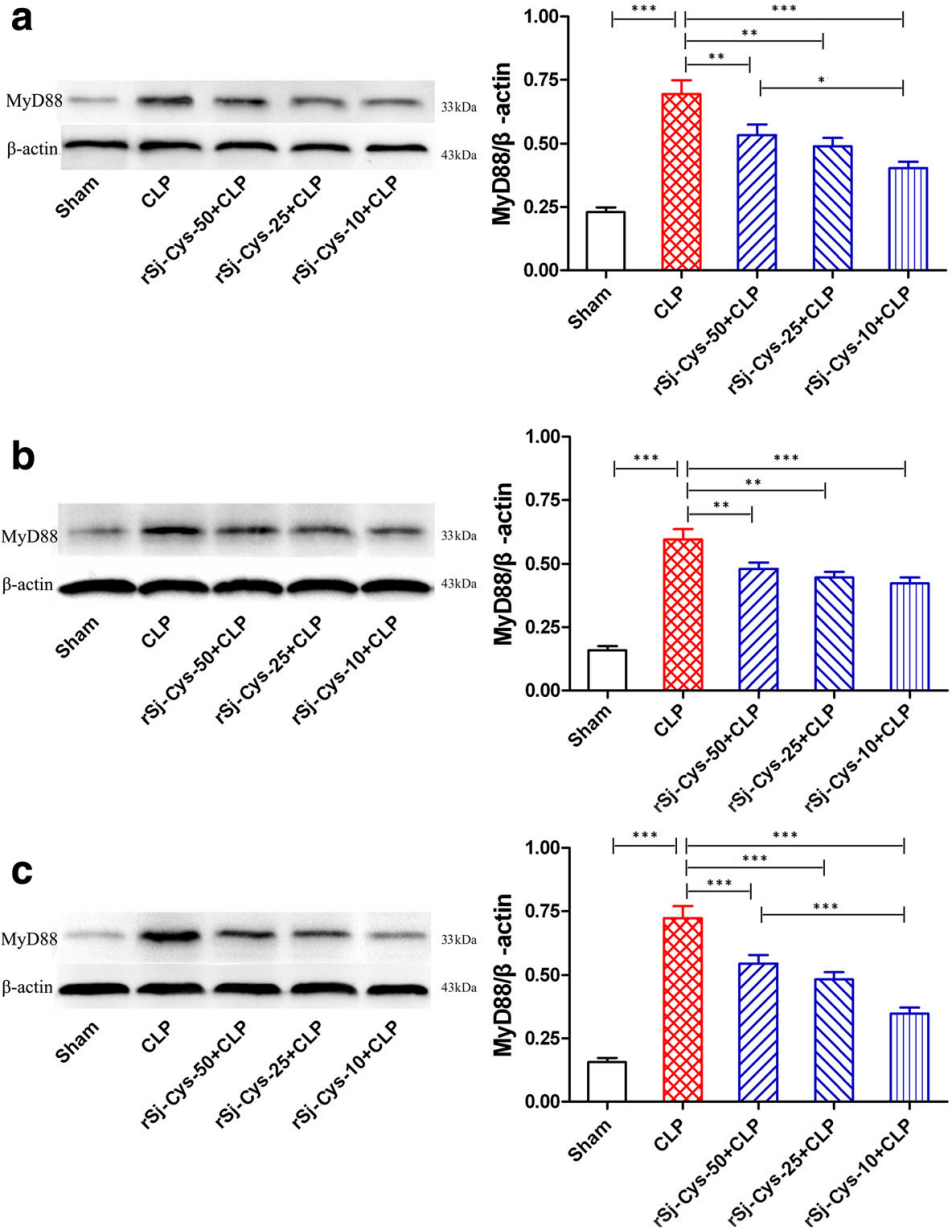
r*Sj*-Cys suppressed the expression of MyD88 in liver (**a**), kidney (**b**) and lung (**c**) of mice with CLP-induced sepsis detected by western blot. The β-actin was detected as control. The density ratio of MyD88/β-actin is shown on the right. The results are shown as the density mean ± SEM for each group (*n* = 6). *P < 0.05, **P < 0.01, ***P < 0.001

MyD88 is the canonical adaptor for inflammatory TLR signaling pathways. Therefore, we detected the expression of MyD88 in the liver, kidney and lung tissues of mice with sepsis treated with or without r*Sj*-Cys to investigate whether r*Sj*-Cys protect major organ with sepsis through inhibiting MyD88 expression. The results demonstrated that the expression of MyD88 in the liver (Fig. 8a), kidney (Fig. 8b) and lung (Fig. 8c) of mice was remarkably increased 12 h after CLP surgery compared to mice with sham surgery (ANOVA: *F*_(4,29)_ = 21.73, *P* < 0.0001; *F*_(4,29)_ = 35.97, *P* < 0.0001; *F*_(4,29)_ = 45.76, *P* < 0.0001, respectively). Treatment with r*Sj*-Cys significantly suppressed the expression of MyD88 in these tissues. r*Sj*-Cys at dose of 10 μg had the better inhibitory effects on the expression of MyD88 than the doses of 50 μg compared to mice with sepsis without treatment in liver (Fig. 8a) and in lung (Fig. 8c). As a control, the level of β-actin was the same in all treated groups (Fig. 8).

## Discussion

Sepsis is still one of the leading causes of mortality in surgical patients or patients in intensive care units with 30–70% mortality for severe sepsis [38]. The superinfection of bacteria in blood leads to the release of large amount of LPS that induces severe inflammatory reaction called systemic inflammatory response syndrome (SIRS) [39]. The out-of-control inflammation eventually results in organ failure and even death. Reduction of pro-inflammatory immune responses during SIRS could improve survival of bacteria-induced sepsis [40]. Previous studies have determined that *S. japonicum*-secreted *Sj*-Cys could stimulate CD4^+^CD25^+^Foxp3^+^ Treg cells [31], alleviate the Th1 dominated immunopathogenesis and actively restrain the colonic inflammation in TNBS-induced experimental colitis in mice [29]. In order to evaluate whether *Sj*-Cys modulates the immune response of mice with sepsis to avoid over-inflammatory reaction to bacterial infection, we first tested if r*Sj*-Cys was able to modulate the functions of macrophages because published evidence showed that macrophages play an important role in inflammation upon infections mainly via secreting inflammatory mediators such as pro-inflammatory cytokines [41, 42]. Gram-negative bacteria release LPS that binds on the TLR4 receptor on macrophages resulting in the release of pro-inflammatory mediators such as TNF-α, IL-6, IL-1β and IL-12 [41, 43]. The murine peritoneal exudate cells (PECs) were collected from donor mice as macrophage source and stimulated by LPS in the presence of r*Sj*-Cys. We observed that r*Sj*-Cys significantly inhibited the LPS-stimulated macrophages to release lower pro-inflammatory cytokines and inflammatory mediator NO in vitro, indicating r*Sj*-Cys has direct inhibitory effects on macrophages’ response agonist LPS. These results in vitro are consistent with those results in vivo showing chronic infection of *Litomosoides sigmodontis* protected mice from bacteremia through inhibiting the level of pro-inflammatory cytokines/chemokines produced by macrophages [44].

Secondly, we treated the mice with CLP-induced sepsis [40] with different dose of r*Sj*-Cys. Strikingly, treatment with r*Sj*-Cys significantly reduced the mortality of mice with CLP-induced sepsis in this study. Mice treated with 10 μg of r*Sj*-Cys protein exhibited 80% survival 96 h after CLP surgery compared to those without treatment that all died 55 h after CLP surgery. Pathological results also showed that sepsis mice treated with 10 μg of r*Sj*-Cys protein remarkably reduced the pathology of important organs (liver, kidney and lung) with much less tissue injury and inflammatory cell infiltration. The therapeutic effects of r*Sj*-Cys treatment were associated with the significant reduction of pro-inflammatory cytokines (TNF-α, IL-1β and IL-6) and boost of IL-10 and TGF-β1 cytokines in sera of mice with sepsis. The results indicate that r*Sj*-Cys is a potent agent in immunomodulating mice immune system to reduce the inflammatory responses possibly through stimulating the regulatory cytokines such as IL-10 and TGF-β1, so as to protect mice with sepsis from over-inflammatory reaction to bacterial infection that eventually leads to serious organ damage or even to death. IL-10 is usually produced by monocytes and Th1 and Th2 lymphocytes with a general inhibitory function to suppress lymphocyte proliferation and cytokine responses. TGF-β1 is produced by most of immune cells including lymphocytes, with various functions to affect T cell proliferation, differentiation and antigen presentation [45]. Regulatory T cells (Tregs) play crucial roles in modulating immune responses mostly through secretion of IL-10, TGF-β1 [14]. Induction of IL-10 has been proven to be essential in the immunomodulation of host immune system that reduce inflammatory responses to helminth infections and to autoimmune/ inflammatory diseases [25–28]. IL-10 has also been proven to be essential in preventing lethal endotoxic shock because depletion of IL-10 resulted in increased mortality in an endotoxemia model [46]. In this study, we identified that treatment with r*Sj*-Cys eventually reduced the expression of MyD88 in liver, kidney and lung tissues. MyD88 is the canonical adaptor for inflammatory TLR signaling pathways [47]. The possible mechanism underlying the therapeutic effect of r*Sj*-Cys is that administration of r*Sj*-Cys stimulates Tregs and/or immune cells to produce regulatory cytokines such as IL-10 and TGF-β1 that inhibit the production of pro-inflammatory cytokines through suppressing TLR-MyD88 activation signal pathway as other helminth-derived proteins did [48–51].

However, in this study the therapeutic effect of r*Sj*-Cys on sepsis does not seem to be dose-dependent. We found that 10 μg of r*Sj*-Cys had the better therapeutic efficacy to reduce the severity of sepsis than the dose of 25 μg or 50 μg of r*Sj*-Cys administrated, which is consistent with the higher levels of IL-10 and TGF-β1 and lower levels of pro-inflammatory cytokines in group of 10 μg r*Sj*-Cys, possibly overdose of r*Sj*-Cys saturates the effect on immune cells or blocks the receptors for other immunomodulatory factors. Another possible reason is that bacteria-expressed r*Sj*-Cys may contain some trace of bacterial components or LPS or other immune enhancers even though the LPS level was under control during production of recombinant *Sj*-Cys (<0.06 EU/ml).

In this study we identified that r*Sj*-Cys owns a therapeutic effect on CLP-induced sepsis through downregulating the TLR adaptor-transducer MyD88 pathway. Other helminth infection or worm-derived materials showed the therapeutic effects on alleviating sepsis through different mechanisms [37, 52–54] such as producing antibody anti-Cyclophilin A, an important inflammatory factor that plays a significant role in the development process of sepsis [53]. Whether other immunological mechanisms or pathways are involved in the protection of *Sj*-Cys against bacteremia-induced excessive inflammation is under investigation.

## Conclusions

*Sj*-Cys, a cysteine protease inhibitor secreted by *S. japonicum*, is a strong immunomodulator that alleviates excessive inflammation caused by bacteremia through stimulating IL-10 and TGF-β1 cytokines and reducing pro-inflammatory cytokines TNF-α, IL-6, IL-1β, possibly acts on macrophages or other effective immune cells via downregulation of the TLR adaptor-transducer MyD88. As a result, treatment with r*Sj*-Cys significantly reduced the multiple organ damage caused by CLP-induced sepsis and provided a therapeutic effect on bacterial sepsis in a mice model.

## Abbreviations

ALT: Alanine transaminase
AST: Aspartate transaminase
BUN: Blood urea nitrogen
CLP: Cecal ligation and puncture
Cr: Creatinine
ELISA: Enzyme-linked immunosorbent assay
IL-10: Interleukin 10
IL-1β: Interleukin 1β
IL-6: Interleukin 6
LPS: Lipopolysaccharides
MODS: Multiple organ dysfunction syndromes
NO: Nitrous oxide
PECs: Peritoneal exudate cells
TGF-β1: Transforming growth factor-β1
TNBS: Trinitrobenzene sulfonic acid.
TNF-α: Tumor necrosis factor alpha
Tregs: Regulatory T cells
